# Structured Psychological Support for people with personality disorder: feasibility randomised controlled trial of a low-intensity intervention

**DOI:** 10.1192/bjo.2020.7

**Published:** 2020-03-02

**Authors:** Mike J. Crawford, Lavanya Thana, Jennie Parker, Oliver Turner, Aidan Carney, Mary McMurran, Paul Moran, Timothy Weaver, Barbara Barrett, Sarah Roberts, Amy Claringbold, Paul Bassett, Rahil Sanatinia, Amanda Spong

**Affiliations:** Division of Psychiatry, Imperial College London, UK; Research and Development, Central & North West London NHS Foundation Trust, UK; Research and Development, Central & North West London NHS Foundation Trust, UK; Division of Specialist Services, Barnet, Enfield & Haringey NHS Trust, UK; Adult Mental Health Directorate, Central & North West London NHS Foundation Trust, UK; Psychiatry and Applied Psychology Department, University of Nottingham, UK; Centre for Academic Mental Health, University of Bristol, UK; School of Health and Education, Middlesex University London, UK; Health Service and Population Research, King's College London, UK; Health Service and Population Research, King's College London, UK; Division of Psychiatry, Imperial College London, UK; Statsconsultancy Ltd, UK; Division of Psychiatry, Imperial College London, UK; Clinical Psychology, Cambridgeshire and Peterborough NHS Foundation Trust, UK

**Keywords:** Personality disorder, psychological treatment, low intensity, brief intervention, randomised trial

## Abstract

**Background:**

National guidance cautions against low-intensity interventions for people with personality disorder, but evidence from trials is lacking.

**Aims:**

To test the feasibility of conducting a randomised trial of a low-intensity intervention for people with personality disorder.

**Method:**

Single-blind, feasibility trial (trial registration: ISRCTN14994755). We recruited people aged 18 or over with a clinical diagnosis of personality disorder from mental health services, excluding those with a coexisting organic or psychotic mental disorder. We randomly allocated participants via a remote system on a 1:1 ratio to six to ten sessions of Structured Psychological Support (SPS) or to treatment as usual. We assessed social functioning, mental health, health-related quality of life, satisfaction with care and resource use and costs at baseline and 24 weeks after randomisation.

**Results:**

A total of 63 participants were randomly assigned to either SPS (*n* = 33) or treatment as usual (*n* = 30). Twenty-nine (88%) of those in the active arm of the trial received one or more session (median 7). Among 46 (73%) who were followed up at 24 weeks, social dysfunction was lower (−6.3, 95% CI −12.0 to −0.6, *P* = 0.03) and satisfaction with care was higher (6.5, 95% CI 2.5 to 10.4; *P* = 0.002) in those allocated to SPS. Statistically significant differences were not found in other outcomes. The cost of the intervention was low and total costs over 24 weeks were similar in both groups.

**Conclusions:**

SPS may provide an effective low-intensity intervention for people with personality disorder and should be tested in fully powered clinical trials.

## Background

People with personality disorder have high levels of mental distress, poor social functioning and reduced quality of life.^1^ One in 20 people have a personality disorder, and as many as half of people in contact with secondary care mental health services meet diagnostic criteria for the condition.^2^ No drugs are licensed for the treatment of the disorder. However, a range of psychological therapies have been developed that improve patient outcomes.^3,4^ Evidence-based psychological treatments for people with personality disorder are intensive and usually involve attending group-based therapy and individual sessions over a 1- to 2-year period.^3^ Limited availability of these treatments mean that many patients do not have access to them, and those that do often face long waiting times before they can start treatment.^5^ Even when these treatments are available, some patients, such as those with coexisting substance dependence, are excluded from them.^6^ Many patients are reluctant to attend groups or are unable to make the commitment required to use intensive treatment, and as many as half of people who are referred to them do not engage with them.^6^ Levels of uptake may be even lower among people from Black, Asian and minority ethnic (BME) communities.^6^ As a result most people with personality disorder do not receive evidence-based psychological treatment.^7^

## Intensity of treatment

Effective brief psychological treatments that do not require attendance at groups have been developed for other mental health conditions, such as anxiety, depression and psychosis. The development of less intensive interventions for people with personality disorder has been hampered by national guidelines for people with borderline personality disorder that caution against the use of interventions lasting less than 3 months.^8^ This recommendation was based on clinical concerns about difficulties that many people with personality disorder have with treatment endings and evidence from a secondary analysis of data from a clinical trial of a brief intervention following self-harm that found higher costs of care among people with personality disorder in the active arm of the trial.^9^

Despite these concerns, services for people with personality disorder are under increasing pressure to treat more patients and ‘stepped care’ approaches have begun to be developed. In stepped care all patients are initially offered a low-intensity intervention and only those who do not respond are offered longer and more intensive treatments.^5,10,11^ Low-intensity interventions are not seen as a substitute for more intensive treatments, but rather an approach that aims to increase access to appropriate care and improve equity of access to more costly and intensive interventions.

Observational studies comparing the impact of lower- and higher-intensity interventions for people with personality disorder report similar outcomes.^10^ However, people are allocated to these interventions on the basis of clinical factors that may affect prognosis, thus biasing estimates of treatment effect. Very few clinical trials of low-intensity interventions for people with personality disorder have been conducted.^12^ Those that have been conducted have required people to attend therapy groups,^13–15^ examined adjunctive interventions designed to enhance the effects of longer-term psychological treatments^16,17^ or have restricted recruitment to subgroups of patients with coexisting conditions.^18^

## Structured Psychological Support

Structured Psychological Support (SPS) for personality and mental health is a low-intensity intervention that was developed in collaboration with people with lived experience.^19^ It involves six to ten sessions of individual psychoeducation and psychological support. SPS is a person-centred approach that recognises the difficulties many people with personality disorder have in trusting others.^20^ During initial sessions, therapists work with patients to formulate a shared understanding of the nature and aetiology of the person's presenting problems and agree on a focus for future sessions. Drawing on techniques used in dialectical behaviour therapy, mentalisation-based treatment and other higher-intensity psychological treatments, therapists work to validate the patient's experiences and use one or more psychological approaches to help them with their most pressing concerns. For many patients this involves trying to find better ways to manage interpersonal problems and strengthening their capacity to tolerate emotional distress. Patients are given self-help materials and information about other forms of community support. Therapists are encouraged to use their experience of what it is like to be with the patient to help guide their understanding of the presenting problems.

## Aims

In this paper, we report findings from a study investigating the feasibility of conducting a randomised trial of SPS versus treatment as usual (TAU) for people with personality disorder.

## Method

### Study design and participants

We conducted a two-arm, parallel group, researcher-masked, randomised controlled trial with a 24-week follow-up assessment (trial registration: ISRCTN14994755).^18^ We included a nested qualitative study, to explore patient and provider beliefs about the impact of the intervention, mechanisms of action and factors that facilitate or hinder its successful delivery.

We recruited participants from community mental health teams across three London boroughs. To take part in the study potential participants had to be aged 18 or over, have a clinical diagnosis of personality disorder and to have provided written informed consent to participate in the study. Written informed consent was obtained from all participants. After obtaining consent, participants completed the Standardized Assessment of Severity of Personality Disorder (SASPD).^21^ A score of eight or more on the SASPD provides a reliable assessment of the likelihood of personality disorder according to ICD-11 criteria. We excluded people who scored less than eight on the SASPD. We also excluded those who had a coexisting diagnosis of an organic or psychotic mental disorder (dementia, bipolar affective disorder, delusional disorder, schizophrenia, schizoaffective disorder or schizotypal disorder), those with cognitive or language difficulties that prevented them from completing study assessments and those who were already receiving psychological treatment for personality disorder.

The authors assert that all procedures contributing to this work comply with the ethical standards of the relevant national and institutional committees on human experimentation and with the Helsinki Declaration of 1975, as revised in 2008. All procedures involving patients were approved by the South Central Research Ethics Committee (Ref 16/SW/0255) prior to the start of data collection.

### Randomisation and masking

Study participants were randomly allocated to SPS or TAU in a 1:1 ratio using an independent web-based service ‘sealed envelope’ (https://www.sealedenvelope.com/simple-randomiser/v1/lists). We stratified randomisation by gender and borough. The randomisation list was encrypted and held in a trial coordinating office preventing access by the research team. The trial manager informed patients, therapists and clinical teams of the allocation status of participants. This information was withheld from researchers who initiated all conversations with patients and staff with a reminder that they needed to remain masked to the allocation arm of participants. When a researcher was inadvertently unmasked arrangements were made for an alternative researcher to undertake follow-up assessments.

### Interventions

Those in the active arm of the trial were offered between six and ten sessions of psychological support. SPS is a person-centred approach that allows therapists to determine the exact number, frequency and duration of sessions based on clinical judgement and patient preference.^19^ During the first two sessions therapists assessed the patient's mental health, personality difficulties and existing understanding of their problems and coping strategies in order to formulate a treatment plan, including a crisis plan. By the end of session two, the participant and the therapist aimed to agree on the focus for the remaining sessions and share this in writing with the participant and their general practitioner. The focus of the remaining sessions depended on the needs and preferences of the participant but included help with developing coping skills, support to better understand problems in relationships or encouragement and advice around the person's social and occupational needs. It could include advice and support to develop skills for emotional regulation, distress tolerance or interpersonal effectiveness.^22,23^

During sessions the therapist discussed the nature of personality disorder, what leads people to develop disturbed interpersonal functioning and what steps people can take to lessen the impact that aspects of their personality can have on their quality of life. SPS draws on the two longer-term evidence-based treatments for people with personality disorder: dialectical behaviour therapy and mentalisation-based treatment. During sessions therapists sought to validate the patient's experience,^24^ and to promote mentalising (the capacity to understand how mental states can affect a person's thoughts, feelings and actions and the thoughts, feelings and actions of others).^25^

Therapists talked to patients about what healthcare services can and cannot do to assist people with personality disorder and supported them to take steps to look after their mental health. Therapists were encouraged to use a range of different methods for communicating with patients according to their preferences, including face-to-face meetings, telephone contacts, texts and emails. Patients were given access to written and/or web-based information and signposted to other services as appropriate. At the end of the sessions participants were generally discharged from secondary care services. This was made explicit to potential participants before they enrolled in the study. However, therapists were able to refer participants to longer-term psychological treatments or continuing care from mental health services if it was judged necessary to do so. Although there was variation in the content and delivery of SPS, all therapists were asked to ensure delivery of five key components:

- information about personality, personality disorder and the role of health services;
- validation and radical acceptance aimed at reducing self-blame and motivating self-efficacy;
- psychological skill(s) for managing the main problem identified by the patient;
- discussion of the role of relationships and structured activities in achieving better mental health;
- use of a ‘mentalising stance’ to highlight the importance of mental states.

All staff delivering SPS were registered mental health professionals with experience of working with people with personality disorder and had completed a 3-day basic training course in mentalisation-based treatment. Staff were given a nine-page guide on the organisation and content of SPS. Staff were asked to attend fortnightly 75 min supervision meetings to discuss their case-load, which was supervised by M.J.C.

Staff delivering SPS were asked to self-complete a proforma for every participant that recorded the number and length of face-to-face, telephone and email/text contacts they had with patients. They were also asked to rate the extent to which they judged they delivered five key components of SPS on a 10-point Likert scale (where zero was not delivered, five was delivered and ten was delivered in full).

TAU was delivered by staff working in community mental health teams. This comprised assessment, care planning and review. Those with coexisting mental health conditions and those judged to be at high risk of severe self-harm may remain under the care of mental health services for a longer period of time. As part of the local care pathway for people with personality disorder, staff discuss the time-limited nature of the service they provide from the outset. Staff delivering TAU were able to refer participants to longer-term psychological treatments in the usual way.

### Measures

At baseline we collected demographic details and assessed eligibility using the SASPD.^21^ Scores on the SASPD range from 0 to 27 with a score of ≥8 indicating probable personality disorder. We also asked participants to complete the International Personality Disorder Examination Screening Questionnaire, a 77-item self-complete questionnaire that provides a reliable indication of specific personality disorders using DSM-IV criteria.^26^

Study participants were asked to complete the following outcome measures at baseline and 6-month follow-up:

- the Work and Social Adjustment Scale (a short validated assessment of social functioning);^27^
- the Warwick and Edinburgh Well-Being Schedule – a seven-item questionnaire that provides a reliable assessment of mental well-being;^28^
- suicidal thoughts using items from the National Household Survey of Psychiatric Morbidity;^29^
- health-related quality of life using the three-level version of EQ-5D (EQ-5D-3L), which is sensitive to change among people with personality disorder;^30^
- satisfaction with care using the four-item Client Satisfaction Questionnaire;^31^
- resource use and costs using a modified version of the Adult Service Use Schedule;^32^
- participants were asked to rate any change in their mental health during the previous 6 months using the Clinical Global Impression Scale (CGI), a seven-point Likert scale from very much improved to very much worse;^33^
- participants were asked to state how confident they are in their ability to ‘get yourself through difficult times and situations’ on a five-point Likert scale (ranging from totally confident to totally unconfident). The psychometric properties of this item have not been tested. It was included in the study following feedback with stakeholders with lived experience of personality disorder.^19^

All outcome measures were assessed again 24 weeks after randomisation. Participants were offered a £20 honorarium for completing the 6-month follow-up interview. Researchers also asked participants about adverse events during all contacts and scheduled visits. All adverse events were recorded from consent until 30 days after the final follow-up interview.

### Collection of qualitative data

We also sought consent to conduct a qualitative interview with up to 20 participants (10 in each arm of the trial). These interviews were conducted by a researcher with lived experience of using services for people with personality disorder and took place after all quantitative data had been collected. During this interview, participants were asked about their experience of taking part in the study and any steps they think we could take to improve the design of a future definitive trial. The interview was semi-structured and guided by a topic guide that was applied flexibly. Participants for the qualitative component of the study were purposively selected to ensure that men and women of different ages and ethnicities were selected from those in both the active and control arm of the trial. We also held a focus group with therapists who delivered SPS. We used a semi-structured topic guide to ask therapists what worked well and less well in the study and explore their views about SPS including the role of the treatment guide, supervision sessions and if/how they felt the intervention worked in practice.

### Data analysis

In keeping with recommendations for feasibility studies, we did not use a power calculation to determine the size of the study. Instead we judged that a total sample of 60 participants would generate sufficient data to assess the rate of recruitment and follow-up and estimate levels of uptake and retention in therapy among those in the active arm of the trial.

Our criteria for determining the success of the feasibility study were: recruitment of at least 48 participants (80% of the target study sample of 60 participants), uptake of SPS by at least 60% of participants in the active arm of the trial, and completion of follow-up interviews at 6 months by 75% of study participants.

As a feasibility trial, the study was not powered to show statistically significant differences between the two arms, hence the primary analyses were descriptive in nature and was focused primarily on characteristics of participants, attrition from the trial, non-adherence to therapy and follow-up. Despite the lack of formal powering, exploratory hypothesis tests were performed to further compare study groups. The analyses were focused on the outcomes measured on a continuous scale. Analyses were performed for the outcomes at 6 months, and also for the change in outcome from baseline to 6 months. All continuous outcomes were found to be approximately normally distributed, and thus the two-sample *t*-test was used for all analyses. The mean difference in outcome between groups was reported, along with corresponding 95% confidence intervals. An additional analysis used the chi-squared test to compare the proportion of patients with an improvement on the CGI at 6 months.

The cost of the SPS intervention was calculated with data from the treatment proformas completed by those delivering SPS and costed using the approach set out by the Personal and Social Services Research Unit.^34^ The cost of TAU was calculated for both randomised groups by allocating a unit cost to all data items that the participants received using data from the Adult Service Use Schedule and nationally available unit costs.^34^

Contemporaneous notes from qualitative interviews were made during each interview and subjected to thematic analysis. We used an inductive approach to analyse the data. The aim was to describe the range of experiences and responses to the intervention, highlighting any patterns and divergences in respondent accounts that support the key research questions relating to feasibility and acceptability of both the intervention and trial design.

### Role of the funder

The funder of the study had no role in study design, data collection, data analysis, data interpretation or writing of the report. The corresponding author had full access to all the data in the study and had final responsibility for the decision to submit for publication.

## Results

**Fig. 1:**
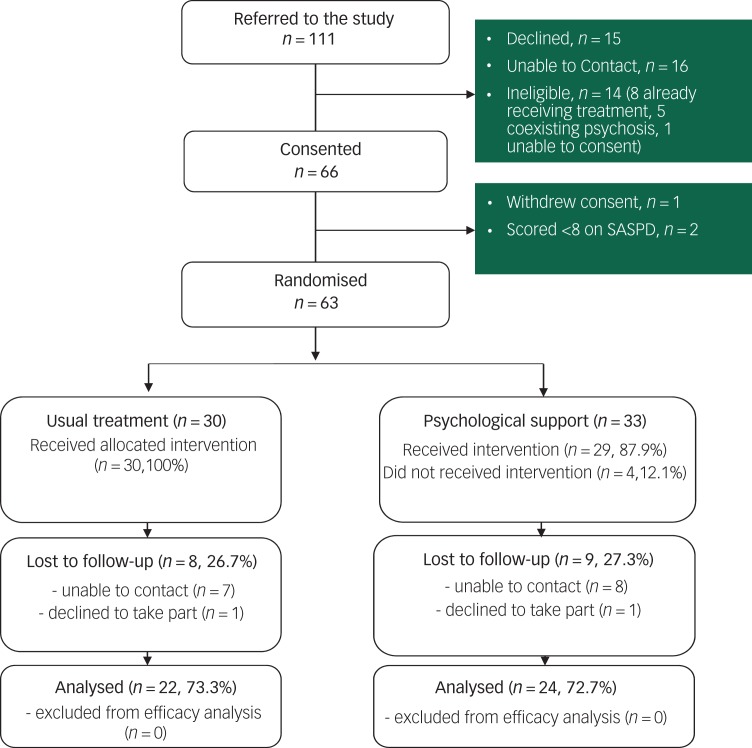
Trial profile.

Between October 2017 and May 2018, 111 patients were referred to the study. We were unable to make contact with 16 (14%) patients, 15 others declined to take part and 14 patients were ineligible (Fig. 1). Of the 63 (57%) who met eligibility criteria and were randomised, 33 were allocated to SPS and 30 to TAU. Six months after randomisation 46 (73%) participants completed a follow-up interview (24, 73% in the active arm of the trial and 22, 73% in the TAU arm).

SASPD, Standardized Assessment of Severity of Personality Disorder

Table 1 summarises the baseline characteristics of participants in the active and control arm of the trial. Study arms were well balanced with the majority of participants in both arms being women, unmarried and not in employment. Mean score on the SASPD was 15.7, indicating moderate/severe personality disorder.^21^

**Table 1:** Patient demographics

Characteristic	Treatment as usual (*n* = 30)	Structured Psychological Support (*n* = 33)
Gender, *n* (%)	30	33
Female	21 (70)	22 (67)
Male	9 (30)	11 (33)
Age, years: mean (s.d.)	37.3 (13.5)	35.4 (9.8)
Marital status, *n* (%)	30	33
Single, never married	21 (70)	26 (79)
Married/partnership	4 (13)	2 (6)
Divorced	4 (13)	4 (12)
Widowed	1 (3)	1 (3)
Employment, *n* (%)	30	33
Employed for wages	6 (20)	6 (18)
Self-employed	0 (0)	2 (6)
Out of work, looking	1 (3)	3 (9)
Out of work, not looking	7 (23)	7 (21)
Homemaker	1 (3)	0 (0)
Student	2 (7)	2 (6)
Retired	1 (3)	0 (0)
Unable to work	12 (40)	13 (39)
Ethnicity, *n* (%)	29	33
White	16 (55)	18 (55)
Asian	0 (0)	2 (6)
Black	3 (10)	2 (6)
Mixed	5 (17)	6 (18)
Other	5 (17)	5 (15)
SASPD score, mean (s.d.)	16.2 (3.9)	15.2 (4.0)
IPDE criteria met, *n* (%)	30	33
Paranoid	26 (87)	26 (79)
Schizoid	24 (80)	23 (70)
Schizotypical	28 (93)	31 (94)
Histrionic	26 (87)	23 (70)
Antisocial	14 (47)	16 (48)
Narcissistic	15 (50)	17 (52)
Borderline	30 (100)	33 (100)
Compulsive	27 (90)	29 (88)
Dependent	23 (77)	27 (82)
Avoidant	26 (87)	31 (94)

SASPD, Standardized Assessment of Severity of Personality Disorder; IPDE, International Personality Disorder Examination.

Participants in the active arm of the trial were collectively offered a total of 252 sessions, and attended 204 (81%) of these. In total, 29 (88%) participants, including 13 (72%) BME participants and 16 (88%) British White and White other participants, attended one or more session of SPS (median 7, range 1–13). Sessions generally lasted 50 min and took place on a weekly or fortnightly basis. Participants received an average of two telephone calls lasting on average 9.4 min. All but five (15%) participants were discharged to primary care after their treatment, with four continuing to receive out-patient follow-up from community mental health teams and one being referred to perinatal mental health services.

Ten therapists delivered the SPS (four mental health nurses, three psychiatrists, two occupational therapists and one psychotherapist). Record of activity forms were received for 27 (93%) of the 29 participants who attended one or more session of SPS. Therapists rated that the five key components of SPS as having been delivered for: information and discussion about personality, personality disorder and the role of health services in 25 patients (93%); validation and radical acceptance in 23 (85%), psychological skills in 20 (74%); discussion of the role of relationships and structured activities in achieving better mental health in 22 (82%); and use of a mentalising stance for 21 (78%).

Baseline and 6-month follow-up scores on continuous study outcomes are presented in Table 2. Levels of impairment in social functioning were lower (mean difference −6.3, 95% CI  −12.0 to −0.6, *P* = 0.03) and levels of satisfaction with care were higher (mean difference 6.5, 95% CI −2.5 to 10.4, *P* = 0.002) among those allocated to SPS than those in the control arm of the trial. At 6-month follow-up 16 (67%) of 24 in the SPS arm of the trial reported improvement on the CGI scale, compared with 10 (45%) of 22 in the control arm of the trial (difference 21%, 95% CI −7% to 49%, *P* = 0.15). High levels of suicidal ideation were reported among participants in both arms of the trial at baseline and follow-up with no differences between group (see Table 3).

**Table 2:** Efficacy outcomes measured on a continuous scale

Outcome and time point	Treatment as usual		Structured Psychological Support		Mean difference^a^ (95% CI); *P*	Effect size, Cohen's *d* (95% CI)
	*n*	Mean (s.d.)	*n*	Mean (s.d.)		
WSAS						
Baseline	30	29.3 (7.7)	33	28.0 (7.5)	–	–
Baseline^b^	22	29.9 (7.6)	24	28.4 (7.7)	–	–
6 months	22	30.9 (8.8)	24	24.6 (10.1)	−6.3 (−12.0 to −0.6); 0.03	0.66 (0.07 to 1.28)
Change^c^	22	1.0 (6.3)	24	−3.8 (8.4)	−4.8 (−9.3 to −0.4); 0.03	0.64 (0.05 to 1.24)
WEMWBS						
Baseline	30	15.1 (4.6)	33	15.3 (3.8)	–	–
Baseline^b^	22	15.3 (4.4)	24	13.8 (3.8)	–	–
6 months	22	15.9 (4.7)	24	17.3 (5.5)	1.4 (−1.6 to 4.5); 0.36	0.27 (−0.31 to 0.85)
Change^c^	22	0.6 (5.0)	24	3.6 (5.2)	2.9 (−0.1 to 5.9); 0.06	0.58 (−0.01 to 1.17)
CSQ-8						
Baseline	30	20.0 (5.8)	33	19.7 (6.0)	–	–
Baseline^b^	21	19.9 (5.7)	22	20.3 (5.2)	–	–
6 months	21	16.0 (5.7)	22	22.5 (6.9)	6.5 (2.5 to 10.4); 0.002	1.02 (0.39 to 1.67)
Change^c^	21	-3.8 (6.0)	22	2.2 (8.1)	6.0 (1.6 to 10.4); 0.008	0.84 (0.22 to 1.46)
ES-visual analogue scale (from the EQ-5D-3L)						
Baseline	30	44.0 (24.0)	33	44.0 (16.0)	–	–
Baseline^b^	22	42.0 (21.0)	24	41.0 (17.0)	–	–
6 months	22	42.0 (26.0)	24	53.0 (26.0)	11 (−4.0 to 26.0); 0.16	0.42 (−0.16 to 1.01)
Change^c^	22	0.0 (22.0)	24	12.0 (22.0)	12 (−1.0 to 26.0); 0.07	0.55 (−0.04 to 1.13)
EQ-5D-3L						
Baseline	30	0.20 (0.35)	32	0.26 (0.36)	–	–
Baseline^b^	22	0.13 (0.34)	23	0.27 (0.39)	–	–
6 months	22	0.23 (0.40)	23	0.36 (0.40)	0.13 (−0.10 to 0.37); 0.28	0.33 (−0.26 to 0.91)
Change^c^	22	0.10 (0.40)	23	0.12 (0.36)	0.02 (−2.1 to 0.25); 0.86	0.05 (−0.53 to 0.64)

WSAS, Work and Social Adjustment Scale; WEMWBS, Warwick and Edinburgh Well-Being Schedule; CSQ, Client Satisfaction Questionnaire; ED-VAS, ED visual analogue scale; ED-5D-3L, three-level version of EQ-5D.

a. Difference calculated as value for Structured Psychological Support minus value for treatment as usual.

b. Summary statistics only for patients with values at 6 months.

c. Change calculated as value at 6 months minus value at baseline.

**Table 3:** Categorical outcomes

Category and time point	Treatment as usual		Structured Psychological Support	
	Total *n*	Outcome, *n* (%)	Total *n*	Outcome, *n* (%)
*Clinical Global Impression*				
Baseline	30		32	
Very much improved		1 (3)		0 (0)
Much improved		7 (23)		4 (13)
Minimal improvement		4 (13)		10 (31)
No change		9 (30)		7 (22)
Minimally worse		3 (10)		2 (6)
Much worse		3 (10)		6 (19)
Very much worse		3 (10)		3 (9)
Baseline^a^	22		23	
Very much improved		1 (5)		0 (0)
Much improved		4 (18)		3 (13)
Minimal improvement		3 (14)		5 (22)
No change		8 (36)		6 (26)
Minimally worse		1 (5)		2 (9)
Much worse		3 (14)		4 (17)
Very much worse		2 (9)		3 (13)
6 months	22		24	
Very much improved		1 (5)		3 (13)
Much improved		1 (5)		7 (29)
Minimal improvement		8 (36)		6 (25)
No change		6 (27)		3 (13)
Minimally worse		0 (0)		2 (8)
Much worse		1 (5)		2 (8)
Very much worse		5 (23)		1 (4)
*Suicidal behaviour*				
Felt life was not worth living				
Baseline	30	21 (70)	33	18 (55)
Baseline^a^	22	16 (73)	24	14 (58)
6 months	22	14 (64)	24	13 (54)
Wished were dead				
Baseline	30	21 (70)	33	18 (55)
Baseline^a^	22	15 (68)	24	13 (54)
6 months	22	13 (59)	24	14 (58)
Thought of taking own life				
Baseline	30	17 (57)	33	17 (52)
Baseline^a^	22	12 (55)	24	13 (54)
6 months	22	10 (45)	23	12 (52)
Attempted to take own life				
Baseline	30	2 (7)	33	0 (0)
Baseline^a^	22	2 (9)	24	0 (0)
6 months	22	1 (5)	24	1 (4)

a. Summary statistics only for patients with values at 6 months

Nine serious adverse events were recorded during the trial; all were admissions to hospital for physical or mental health conditions. In addition, seven other adverse events were recorded, one of which (self-neglect following the end of active treatment) was thought to be related to the trial. The cost of the SPS was on average £360 per participant, of which £317 was the cost of the one-to-one therapy and £67 was the cost of telephone calls. The use and cost of medication, hospital and community services was similar in both groups.

Qualitative data provided support for the role that SPS could play in improving the mental health of people with personality disorder. Participants who were offered active treatment felt it was helpful to think about alternative ways of managing difficult situations and considering the perspectives of others. Participants also reported that they valued receiving one-to-one sessions and being offered choices about the duration and frequency of sessions. They reported receiving support that was non-judgemental. However, some wanted to know more about the intervention before it started and would have liked to have been given written information. Some participants said that they would have liked to have had further follow-up sessions with one stating the ten sessions they received was ‘just not enough’ and another stating that the six sessions they received did not ‘scratch the surface’. One participant stated that they were concerned that the intervention was a ‘cost-saving measure’ designed to ensure they were discharged from services. Participants had a range of different experiences of therapists that included feeling supported and listened to, whereas others reported feeling dismissed and patronised.

Participants in the TAU arm of the trial expressed concerns about what they were offered, including little contact with services and long waiting times to access other sources of help. Participants in both arms of the study liked the written communications they received from the study team and several thought that it would have been helpful to have more frequent contact with the study researchers. Most participants thought that study questionnaires were easy to respond to and asked about things that mattered to them, but two felt that the available responses restricted what they wanted to say. Although participants said that the process of randomisation had been explained to them, many found the gap between completing the initial assessment and finding out which group they had been allocated to was anxiety-provoking and others questioned whether they really had been allocated to interventions based on chance.

Nine of the ten therapists that delivered SPS took part in a focus group, and one therapist took part in a semi-structured interview. Staff delivering SPS spoke of their surprise at how much ‘meaningful work’ it had been possible to do in a time-limited number of sessions. Staff enjoyed having the flexibility of the approach that had enabled them to deliver patient-centred care and reported that it had been helpful to have a ‘buffet’ of approaches and techniques they could draw on during the sessions. Staff felt that it had been possible for them to manage patient expectations about the short-term nature of the intervention and highlighted the importance of group supervision as a means of shared learning.

## Discussion

### Main findings

These results demonstrate that a randomised trial of SPS for personality and mental health is entirely feasible. We were able to recruit to target and levels of engagement in the active intervention were high. We followed up 73% of the participants at 6 months, slightly fewer than our 75% target.

The prevalence of personality disorder among people using mental health services is high. The reluctance of many patients to talk about their problems in groups and limited availability of long-term therapies meant that clinicians found it easy to identify potential participants. Entry criteria for the trial were broader than those of high-intensity psychological treatments. We accepted patients with coexisting alcohol and drug dependence who would not have been considered suitable for group-based psychotherapy. Because groups take place at regular times some patients with work, child care or other regular commitments may be unable to attend them. In contrast, therapists and patients were able to schedule sessions of SPS around these commitments. As a result, we found it easy to recruit to target. The main limit on the number of people we could recruit was the availability of staff to deliver the intervention.

Levels of uptake of SPS in the active arm of the trial were high, with 29 (88%) attending one or more session and a median attendance of seven sessions. There was greater representation of BME patients in the study than among people taken on by local high-intensity treatment services for people with personality disorder by secondary care services. Ratings made by therapists delivering SPS indicated that in over 70% of cases it was possible to deliver the key components of the intervention within the number of sessions that participants attended.

Quantitative data collected from study participants demonstrated the potential of this low-intensity intervention to benefit people with personality disorder. Although the study was not powered to detect statistically significant differences in study outcomes, those in the active arm of the trial reported a significant improvement in social functioning and greater satisfaction with care at the 6-month follow-up. Furthermore, although not statistically significant, the data also suggested that SPS has a potentially positive impact on mental health and health-related quality of life. The potential for the active intervention to improve social functioning among people with personality disorder is important because, although most people with the condition report improved mental health over time, impairment in social functioning is considered to be an enduring feature of the condition.^35^ We estimate that the cost of delivering the intervention was modest, but cost-effectiveness remains to be tested.

Qualitative data from study participants provided additional support for the benefits that some people with personality disorder may gain from SPS. Study participants valued the choices they were offered and the flexibility around the timing, frequency and content of their sessions.

### Study design

Our data also suggest ways that the design of the study could be improved. Many of those who were allocated to TAU spoke of the upset they experienced when they found out that they would not be offered SPS. People in the TAU arm of the trial often felt unsupported with the service they received. Resentful demoralisation associated with allocation to control treatment is an issue in many clinical trials.^36^ Future trials of low-intensity interventions for people with personality disorder should supplement TAU with some form of additional support. This could help ensure that any differences seen in outcomes are not influenced by the responses people may have to their allocation status. Current national guidelines state that all people with borderline personality disorder should be offered support to develop a crisis plan,^8^ but a recent national audit found that half of people did not have such a plan.^37^ A clinical trial of jointly developed crisis plans with people diagnosed with borderline personality disorder found that, although many patients valued them, they did not lead to measurable changes in patient outcomes.^38^ Hence, the addition of a session to develop a joint crisis plan could ensure that all study participants receive additional support while still enabling a valid assessment of any patient benefit associated specifically with SPS.

Participants in the active arm of the trial told us that they would have liked to have been given more information about SPS before their first session. It would be a simple step to add an information sheet for patients in advance of the start of SPS that could be discussed with the therapist at the start of the sessions.

People in the active arm of the trial also asked if it would be possible to have additional sessions of SPS. Although it would be possible to extend the number of sessions that patients are offered this could undermine the rationale for this low-intensity intervention. It is not unusual for people who are completing high-intensity psychological interventions to want more time in treatment.^6^ For people with long-term mental health conditions who can struggle with endings, worries about the ability to cope without ongoing support are well grounded. One of the core aims of many interventions for people with personality disorder, including SPS, is to try to help people develop greater self-confidence in coping with difficult feelings and situations. Some psychological interventions include a limited number of follow-up sessions in which people are able to meet with a therapist sometime after regular treatment has finished.^39^ Consideration could be given to adding one or more top-up sessions to this intervention.

### Limitations

Limitations of the study include length of follow-up, the level of drop-out and the process used for assessing treatment fidelity. Personality disorder is a long-term mental health condition and it is therefore important to examine the long-term impact of interventions. The follow-up period in any explanatory trial of SPS should therefore be longer than 6 months. The follow-up rate in the trial was slightly lower than the 75% target. Further attention would need to be paid to improving the rate of follow-up in a future trial. Given feedback from study participants that they would have liked additional contact with study researchers, additional points of contact could be built into the protocol of a future study.

We relied on self-ratings of therapists to explore the feasibility of delivering SPS. The results of this exercise suggested that it was possible to deliver the key components of SPS within the available sessions. Previous studies of psychological interventions for people with personality disorder have examined treatment fidelity more objectively by recording sessions and obtaining independent ratings of treatment fidelity. Such an approach should be considered in any full-scale trial of this intervention.

We did not use a formal diagnostic interview to confirm the diagnosis of personality disorder in this trial. Such interviews are lengthy, often taking an hour or more to complete. Data from a previous trial of problem-solving therapy for people with personality disorder that used a structured diagnostic interview to assess eligibility for trial inclusion found that nearly all those referred to the trial (650, 95.3% of 682) met criteria for personality disorder,^15^ showing that clinicians referring to the trial were able to accurately identify relevant patients. Time-consuming structured assessments may, therefore, be unnecessary, particularly where the specific type or types of personality disorder are not pertinent. With a mean SASPD score of 15.7, participants in this trial had a level of severity far in excess of the threshold for moderate personality disorder and we can be confident that study participants would have had their clinical diagnosis confirmed had they taken part in a lengthier interview. Structured diagnostic interviews are not used in clinical practice and may not be appropriate in the context of pragmatic clinical trials that aim to generate knowledge that can be applied in ‘real-world’ settings.

We do not know whether the benefits participants in the active arm of the trial reported were the result of the specific factors that make up SPS, or to non-specific factors related to time spent with a therapist. However, the negative results of a recent, fully powered, trial of problem-solving therapy for people with personality disorder suggest that time spent with therapists alone is not sufficient to improve patient outcomes.^15^

This study was conducted in a secondary care mental health setting, however, many people with personality disorder are managed within primary care. Future research should examine the acceptability and effectiveness of SPS delivered in primary care settings.

### Implications

The main implication of this study is that it is feasible to conduct a clinical trial of SPS for people with personality disorder. A future trial should be sufficiently large to assess minimum clinically important differences between study arms. Consideration should be given to providing study participants with more regular contact with researchers and enhancing TAU for those in the control arm of the trial. Should a low-intensity intervention such as SPS be found to improve the health and social outcomes of people with personality disorder it has the potential to be incorporated into a stepped-care approach for people with this condition. This could lead to more patients being offered evidence-based care than is currently the case and high-intensity treatments being reserved for those who most need them.

## Data Availability

All data requests should be submitted to the corresponding author for consideration. Access to anonymised data may be granted following review.
